# Functional consequences of a rare human serotonergic 5-HT_1A_ receptor variant

**DOI:** 10.3389/fphar.2023.1270726

**Published:** 2023-09-18

**Authors:** Merav Tauber, Yair Ben-Chaim

**Affiliations:** Department of Natural Sciences, The Open University of Israel, Ra’anana, Israel

**Keywords:** G protein coupled receptors, serotonin receptors, genetic variation, voltage dependence, *Xenopus* oocytes

## Abstract

Serotonin (5-HT) plays a central role in various brain functions via the activation of a family of receptors, most of them G protein coupled receptors (GPCRs). 5-HT_1A_ receptor, the most abundant 5-HT receptors, was implicated in many brain dysfunctions and is a major target for drug discovery. Several genetic polymorphisms within the 5-HT_1A_ receptor gene were identified and linked to different conditions, including anxiety and depression. Here, we used *Xenopus* oocytes to examine the effects of one of the functional polymorphism, Arg220Leu, on the function of the receptor. We found that the mutated receptor shows normal activation of G protein and normal 5-HT binding. On the other hand, the mutated receptor shows impaired desensitization, probably due to impairment in activation of β arrestin-dependent pathway. Furthermore, while the 5-HT_1A_ receptor was shown to exhibit voltage dependent activation by serotonin and by buspirone, the mutated receptor was voltage-independent. Our results suggest a pronounced effect of the mutation on the function of the 5-HT_1A_ receptor and add to our understanding of the molecular mechanism of its voltage dependence. Moreover, the findings of this study may suggest a functional explanation for the possible link between this variant and brain pathologies.

## Introduction

The neurotransmitter serotonin (5-hydroxytryptamine, 5-HT) activates a family of receptors comprised of seven subfamilies, six of them are G protein coupled receptors (GPCRs) and one is ligand gated ion channel (5-HT3) (Nichols and Nichols, 2008). 5-HT receptors mediate many cellular processes (Seyedabadi et al., 2014; Sharp and Barnes, 2020) and have been implicated in many brain disorders, including depression and anxiety (Švob Štrac et al., 2016; Carhart-Harris and Nutt, 2017). 5-HT_1A_ receptor is the most abundant serotonin receptor and its ligands serve as pharmacological target for antidepressants and antipsychotic drug discovery (Bantick et al., 2001; Newman-Tancredi, 2010; Powell et al., 2022; Yao et al., 2022). 5-HT_1A_ receptors regulate serotoninergic neurotransmission in serotoninergic neurons in the raphe nuclei where they serve as autoreceptors (Lanfumey and Hamon, 2000). In addition, 5-HT_1A_ are expressed in several regions, including the hippocampus, the amygdala and the septum, where they are expressed post-synaptically (Li et al., 2006; Carhart-Harris and Nutt, 2017). 5-HT_1A_ receptors are predominately Gi/o-coupled receptor. As such, they inhibit adenylyl cyclase and activate inwardly rectified potassium channels, thus causing cell hyperpolarization (Carhart-Harris and Nutt, 2017).

Genetic variation in the human 5-HT_1A_ receptor may affect the function of the receptor, as well as its pharmacological properties. Therefore, such genetic variations may be related to some neuropsychiatric disorders. Moreover, variation in the receptor may alter the potency of ligands that are used clinically (Brüss et al., 1995; Göthert et al., 1998; Wu and Comings, 1999; Li et al., 2006). Several genetic polymorphisms were identified for the 5-HT_1A_ receptor. The most investigated 5-HT_1A_ receptor variants is the change of nucleotide C to G, C (-1019)G (rs6295), which is located in the gene promoter and is quite prevalent in the common population (Wu and Comings, 1999). In addition, three functional polymorphisms within the coding region were identified: Ile28Val (rs1799921) and Gly22Ser (rs1799920) and Arg219Leu (rs1800044) (Göthert et al., 1998).

Previous studies have shown that the binding of ligands to the naturally occurring Ile28Val variant did not differ from that observed of the wild-type (wt) receptor (Brüss et al., 1995). Furthermore, similar results were obtained for the Gly22Ser variant, although in this case a decreased agonist-induced desensitization was reported for this variant (Rotondo et al., 1997).

The latter variant, Arg219Leu (later denoted as Arg220Leu; we will use this term here), was first identified in a patient suffering from Tourette’s syndrome (Erdmann et al., 1995) and was later linked to major depression (Haenisch et al., 2009). This variant contains a missense mutation in the third intracellular loop of the receptor, a region implicated in effector coupling of GPCRs. (Gether, 2000; DeVree et al., 2016). Previous functional study found that the Arg219Leu variant is expressed normally in HEK293 cells (Brüss et al., 2005), suggesting that this variant does not affect the stability of the receptor or its ability to be translated and reach the plasma membrane. Furthermore, this variant apparently does not change the binding properties of 5-HT_1A_ receptor. On the other hand, the same study found that the ability of the mutated receptor to inhibit forskolin-induced cAMP accumulation was impaired, suggesting an impairment in G protein activation (Brüss et al., 2005).

In recent years, the modulation of GPCRs activity by membrane potential has emerged as a new signaling paradigm. Several studies employed different approaches to show that membrane potential directly modulate the affinity and activity of many GPCRs, including receptors for acetylcholine (ACh) (Ben-Chaim et al., 2003; Ben-Chaim et al., 2006; Rinne et al., 2015), glutamate (Ohana et al., 2006), dopamine (Sahlholm et al., 2008a; Sahlholm et al., 2008b; Ågren and Sahlholm, 2020), adrenaline (Rinne et al., 2013; Birk et al., 2015), purines (Martinez-Pinna et al., 2004; Martinez-Pinna et al., 2005) opioids (Ruland et al., 2020) and prostanoids (Kurz et al., 2020). Recently we found, that the 5-HT_1A_ receptor is voltage dependent as well, as the potency of 5-HT to activate this receptor to the receptor is stronger under hyperpolarization than under depolarization (Tauber and Ben Chaim, 2022). The molecular mechanism that underlies the effect of membrane potential on GPCRs is not fully understood (David et al., 2022). For muscarinic receptors and glutamatergic receptors, it has been suggested that the G protein coupling site may play a role in the voltage-dependence of agonist binding (Ben-Chaim et al., 2006; Ohana et al., 2006). Specifically, mutating 5 residues in the N terminal of the third intracellular loop of the M2 muscarinic receptor (M2R) abolished the voltage dependence of ACh binding to this receptor. As the Arg220Leu variant is mutated in this region of the 5-HT_1A_ receptor, we sought to study the effect of this mutation on its voltage dependence.

## Materials and methods

### Preparation of cRNA and oocytes

The following cDNA plasmids were used in this study: The 5-HT_1A_ receptor (kindly provided by Dr. Erhard Wischmeyer from the University of Wurzburg, Germany) (Renner et al., 2012), the β-arrestin2 and G protein receptor kinase 3 (GRK3) (kindly provided by Dr. Abraham Kovoor, from the University of Rhode Island, United States), the two subunits of the G protein activated inward rectifying K^+^ (GIRK) (GIRK1 and GIRK2) and the α subunit of the G-protein (Gαi3).

All cDNA plasmids were linearized with the appropriate restriction enzymes (Tauber and Ben Chaim, 2022). The linearized plasmids were transcribed *in vitro* using the mMESSAGE mMACHINE™ Transcription Kit (Invitrogen, Waltham, MA, United States).

*Xenopus laevis* oocytes were isolated and incubated in NDE96 solution composed of ND96 (in mM: 96 NaCl, 2 KCl, 1 CaCl2, 1 MgCl2, 5 Hepes, with pH adjusted to 7.5 with NaOH) with the addition of 2.5 mM Na + pyruvate, 100 units/mL penicillin, and 100 μg/mL streptomycin (Tauber and Ben Chaim, 2022). Oocytes were injected with cRNAs of 5-HT_1A_ receptor (2 ng) and GIRK1 and GIRK2 (200 pg each). In addition, cRNA of Gαi3 (1,000 pg) was injected to improve the relative activation by the agonist (Peleg et al., 2002).

Chemicals were purchased from Sigma Israel (Rehovot, Israel).

### Current measurements

The currents were measured 3–6 days after cRNA injection using the standard two-electrode voltage clamp technique (Axoclamp 2B amplifier, Axon Instruments, Foster City, CA, United States or OC-725, Warner Instruments, Hamden, CT, United States). Oocytes were placed in a 200 µM recording bath containing ND96 solution and recording was performed with two electrodes pulled from 1.5-mm Clark capillaries (CEI, Pangboure, England). Both electrodes were filled with 3 M KCl solution and the electrodes resistances were 1-5 MΩ. The 5-HT-mediated GIRK currents were measured in a 24 mM K^+^ solution (in mM: 72 NaCl, 24 KCl, 1 CaCl2, 1 MgCl2, 5 Hepes, with pH adjusted to 7.5 with KOH) (Friedman et al., 2020). The bath was perfused constantly with either solution in a perfusion rate of∼2 mL/min pCLAMP10 software (Axon Instruments) was used for data acquisition and analysis.

### Data analysis

The dose response curves were fitted by the following equation 1:

$$Y=Bottom+\left(X^{Hill\;slope}\right)*\left(Top-Bottom\right)/\left(X^{Hill\;Slope}+{EC50}^{HillSlope}\right)$$ (1)

In this equation, *Y* is the normalized response, *X* is the agonist concentration, Hill slope is the slope factor and EC_50_ is the agonist concentration that evokes the half-maximal response.

The time constant of the decay of 5-HT_1A_ receptor-activated GIRK currents was extracted by fitting a single exponential to the decay of the current from the time the current has declined to 80% of its maximal level to the time it reached a plateau (Ben Chaim et al., 2013).

### Statistical evaluation

Statistical analysis was conducted using Prism GraphPad software. Significance was evaluated by Student’s two-tailed *t*-test. Estimating the difference between the EC_50_ values was conducted by the extra-sum-of-squares F test.

## Results

To characterize the 5-HT_1A_ (Arg220Leu) variant, The human 5-HT_1A_ was point mutated at this position and oocytes were injected to express the following proteins involved in the signal transduction leading to activation of K^+^ currents by the receptor via the G-protein βγ subunits: The wt and mutated 5-HT_1A_ receptor, the two subunits of the GIRK channel (GIRK1 and GIRK2), and the Gαi3 subunit (Ben-Chaim et al., 2003; Friedman et al., 2020).

**FIGURE 1:**
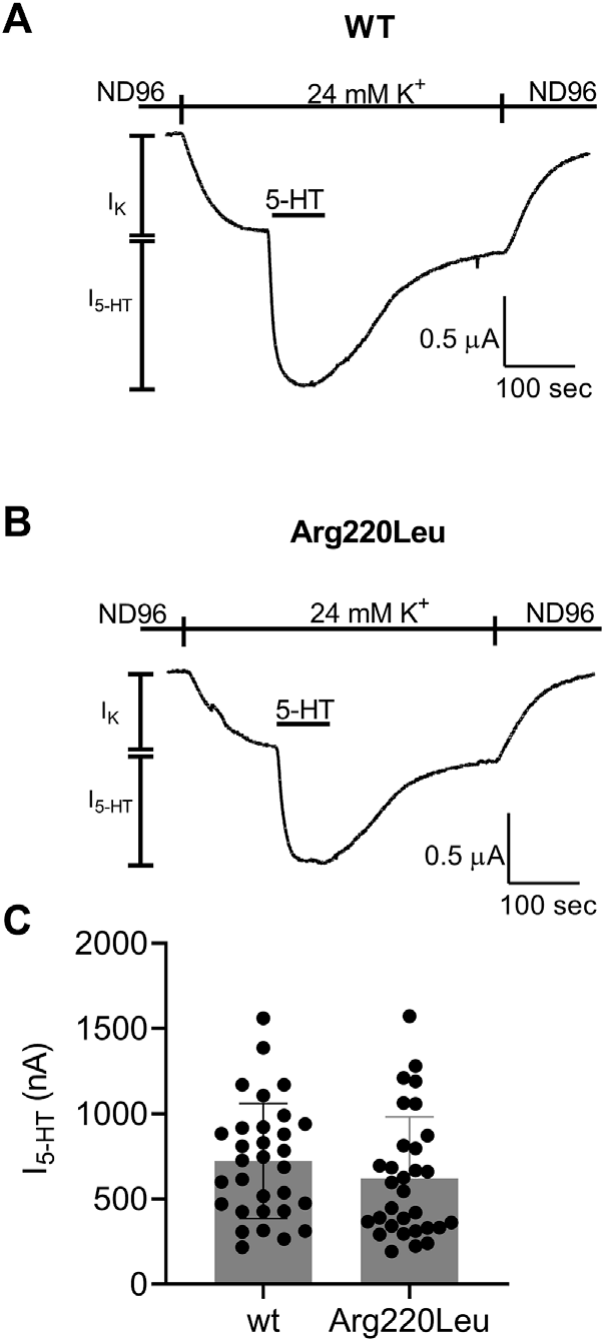
Activation of GIRK channels by wt and Arg220Leu 5-HT_1A_ receptors **(A, B)** Examples of recordings from wt and mutated receptor expressing oocytes in response to application 5000 nM 5-HT at the indicated time **(C)**. Collected results. Each circle represent the amplitude of I_5-HT_ measured from one oocytes. The mean ± SD is shown as horizontal lines.

It has been reported that the Arg220Leu variant exhibits impaired G protein activation. To verify this observation in our functional expression system we compared the currents evoked by 5 µM 5-HT in oocytes expressing the wt 5-HT_1A_ receptor to currents evoked under the same conditions in oocytes expressing the Arg220Leu mutant. Figures 1A, B depict examples for recordings from wt and mutated receptor-expressing oocytes, respectively. In each experiment, the oocyte was voltage-clamped to −80 mV, in a low K^+^ (2 mM K^+^) solution, ND96 (see Materials and Methods). Replacement of the ND96 by the 24 mM K^+^ solution evoked a basal GIRK current (I_K_). Then, 5-HT was applied, evoking further agonist-induced current, I_5-HT_. I_5-HT_ was terminated upon washout of 5-HT. The cumulative results (Figure 1C) show that in our functional assay the Arg220Leu mutant activates the G protein to nearly the same extent as the wt 5-HT_1A_ receptor. The mean current amplitudes (±SE) were 724 ± 330 nA in wt expressing oocytes and 622 ± 360 nA in Arg220Leu mutant expressing oocytes (N = 31 in each group. The two groups are not significantly different, student unpaired *t*-test, *p* = 0.24). This is in contrast to the previously reported effect of this mutation on the inhibition of cAMP accumulation.

**FIGURE 2:**
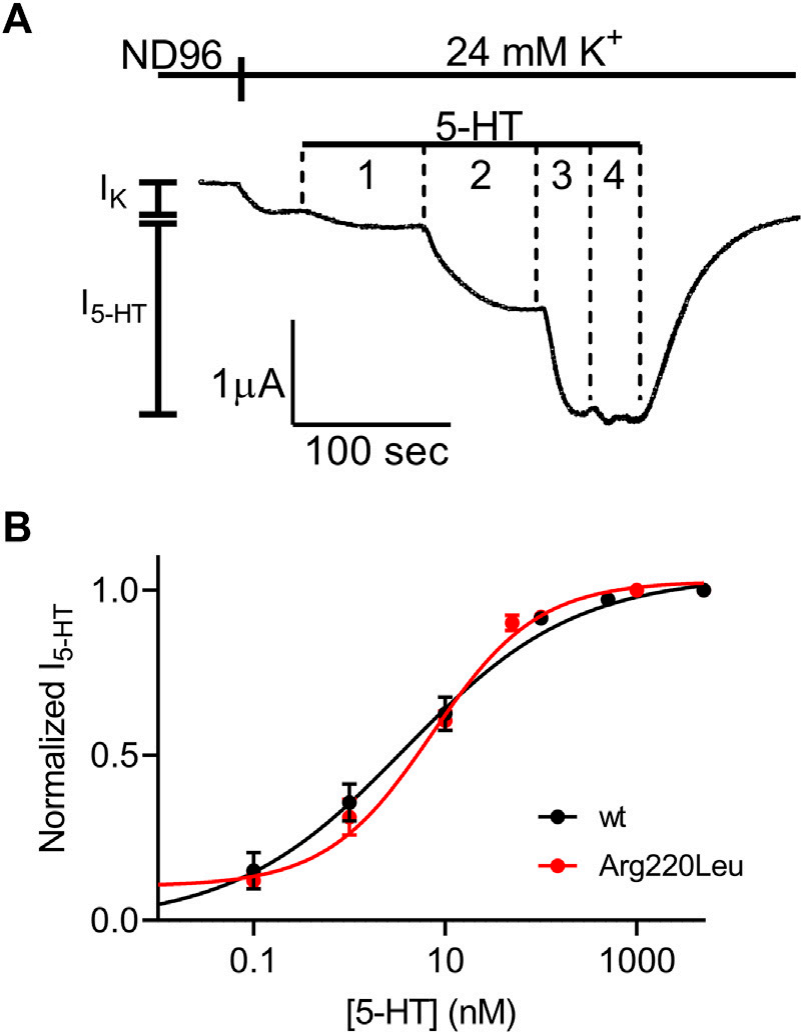
Dose-response relation of 5-HT_1A_ receptor activated GIRK currents **(A)** Measurement of the relationship between 5-HT concentration and Arg220Leu receptor activated GIRK currents at −80 mV. Basal GIRK current evolved following replacement of the solution to a high K^+^ solution. Then, 4 different 5-HT concentrations were applied (0.1, 1, 10, 100 and 5,000 nM, numbered 1-4) and the response for each concentration was measured **(B)** Dose response curves for wt 5-HT_1A_ receptor (black; Taken from Tauber and Ben-Chaim, 2022) and Arg220Leu receptor (red). The responses were normalized to the response evoked by 5000 nM 5-HT. Each point represents the mean (±SEM) from 13-27 oocytes. The solid black and red lines were generated by fitting equation 1 to the data (see Materials and Methods). The EC_50_ values obtained for the two graphs were not significantly different (*p* = 0.55).

We next evaluated the potency of 5-HT to activate the mutated receptor. To this end, we repeated the experiment described in Figure 1 with several 5-HT concentrations. An example for such an experiment is depicted in Figure 2A where the activation of 5-HT induced GIRK currents in response to four concentrations of 5-HT, applied sequentially, was measured. To be able to compare results from different oocytes, I_5-HT_ evoked by any 5-HT concentration was normalized to I_5-HT_ evoked by a saturating 5-HT concentration (5 µM; higher 5-HT concentration did not evoke higher GIRK currents) in the same oocyte. By combining the results of experiments from 8 batches of oocytes we constructed a full dose response curve (Figure 2B) and compared it with similar curve constructed from wt receptor (Figure 2C, black, taken from (Tauber and Ben Chaim, 2022)). To evaluate the effect of the mutation on the potency of 5-HT, the curves were fitted to Eq. 1 and EC_50_ value of 6.7 nM was extracted for the mutated receptor. This value was comparable to the EC_50_ value of 3.8 nM extracted from the curve obtained for the wt receptor (Tauber and Ben Chaim, 2022) (the two EC_50_ values are not significantly different, extra-sum-of-squares F test, *p* = 0.55), suggesting that the mutation had only a minor effect on the potency of 5-HT in activating the receptor.

**FIGURE 3:**
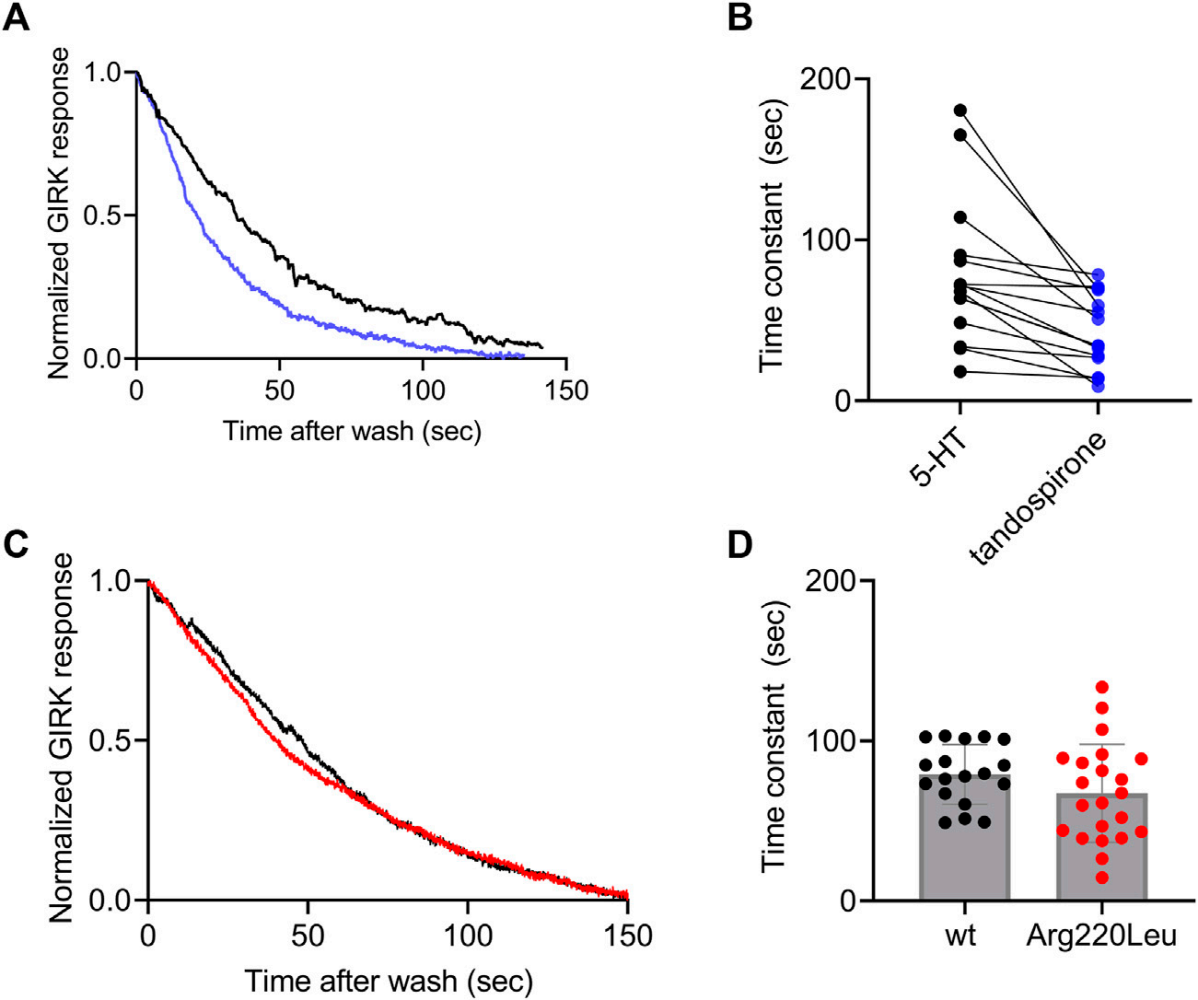
Measurement of the dissociation of agonist from the 5-HT_1A_ receptor by measuring the deactivation of receptor-evoked GIRK current following agonist washout **(A)** A comparison of the decay of GIRK currents evoked by 5-HT (black) or tandospirone (blue) following washout of the agonist at time zero. To enable comparison between the two recordings the currents were normalized by setting the value in each recording where the current reached 80% of its maximal amplitude as 1 **(B)** Results from 15 oocytes subjected to both agonists. Each two dots connected with a line represent one oocyte. The time constant of the decay of 5-HT-evoked currents is significantly higher than that of tandospirone-evoked currents (*p* = 0.001) **(C)** A comparison of the decay of GIRK currents evoked by 5-HT in oocytes expressing wt receptor (black) or Arg220Leu (red) following washout of the agonist at time zero. The currents were normalized to enable comparison between the two recordings **(D)**. Collected results from 18 oocytes expressing wt receptor and 18 oocytes expressing Arg220Leu. The time constant of the decay of 5-HT-evoked currents is not significantly different between the two groups (*p* = 0.63).

It has been reported for other GPCRs that the rate of the decay of agonist-induced GIRK currents upon washout of the agonist may serve as a measure of ligand dissociation from the receptor. Specifically, we showed in the M2R, the CB1 receptor and the mGluR3, that the dissociation rate of the agonist from the receptor (Ohana et al., 2006; Ben Chaim et al., 2013; Goldberger et al., 2022). Can be evaluated from the decline of agonist-induced GIRK currents following the washout of the agonist. To determine whether this holds also for 5-HT_1A_ receptor-activated GIRK currents, we measured the dissociation GIRK currents following the washout of the two agonists that widely differ in their affinities toward the receptor, 5-HT and tandospirone (Tauber and Ben Chaim, 2022). We expect that if the decline of the GIRK currents indeed reflects the dissociation of the agonist from the receptor, then that the measured decline will be differ between the two agonists. If some other downstream process dictates the decline of the currents, then the measurements are not expected to be contingent on the agonist used. To examine this prediction, we measured the deactivation time of the 5-HT or tandospirone receptor-activated GIRK currents. To this end oocytes expressing the 5-HT_1A_ receptor and the GIRK channel were voltage clamped and were subjected to either 5-HT or tandospirone. After the agonist-induced current reached a plateau, the agonist was washed out by perfusion with an agonist-free solution. Agonist application and washout were completed within 5 s (Ben Chaim et al., 2013). Agonist washout resulted in the decline of the receptor-induced GIRK current declines. Then, the experiment was repeated with the second agonist. An example of normalized current decay measured from the same oocyte following washout of either 5-HT (black) or tandospirone (blue) is shown in Figure 3A. From such experiments, the time constant of the decay was extracted by fitting a single exponential equation to the decay. To avoid the possibility that of re-association of the agonist to the receptor during the washout may affect the results we started the fit only after the current decayed to 80% from its maximal amplitude, when no residual agonist is expected to be present. The results (Figure 3B) show that the time constant of 5-HT dissociation is significantly slower than that of tandospirone in all oocytes tested (N = 15; *p* = 0.01, paired *t*-test). These results suggest that the decline rate of the GIRK current depends on the affinity of the agonist. Therefore, this parameter may be used as a measure for agonist dissociation rate.

Next we measured the dissociation time constants of 5-HT from wt receptor and from the Arg220Leu mutant. Examples of normalized currents decay are shown in Figure 3C. As seen from these recordings and from the collected results (N = 18 for wither wt and Arg220Leu), the dissociation time constants were not significantly different between wt receptor (79.1 ± 17.6 s) and the Arg220Leu mutant (75.4 ± 27.4 s; *p* = 0.63, unpaired *t*-test), consistent with the similar EC_50_ values reported above (Figure 2).

**FIGURE 4:**
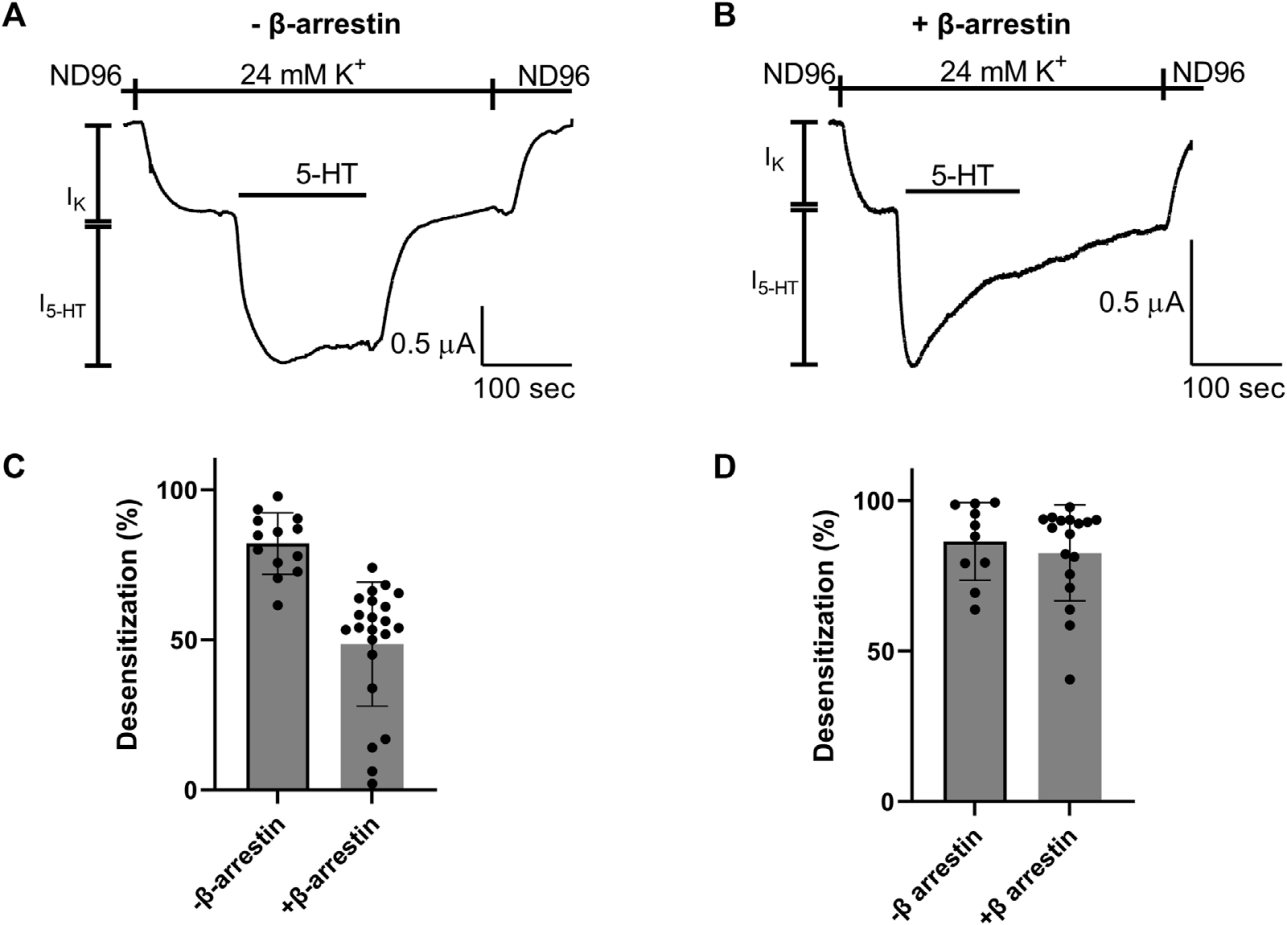
Effect of Arg220Leu mutation on β arresting signaling **(A, B)**. Examples of recordings of I_5-HT_ following long application of 5-HT from oocytes expressing the wt 5-HT_1A_ receptor without co-expression of β arresting and GRK3 **(A)** and with the co-expression of these proteins **(B–D)**. The mean desensitization values obtained from traces such as shown in panels A and **(B)** A significant effect of β-arrestin and GRK3 expression was observed in wt receptor expressing oocytes (*p* < 0.0001) **(C)**, but not in oocytes expressing the Arg220Leu mutant (*p* = 0.52) **(D).**

We next asked whether the Arg220Leu mutation affected the ability of the receptor to activate other signaling pathways. As GPCRs activate both G protein mediated pathways and G protein independent pathways, we focused on the β-arrestin signaling pathway. To this end we co-expressed in the oocytes the β-arrestin and G protein kinase 3 (GRK3). It was shown before for other GPCRs, that such co-expression results in a functional system where the activation of β-arrestin signaling pathway could be evaluated from the level of desensitization of GPCR induced GIRK currents (Jin et al., 1999; Rozenfeld et al., 2021). Figures 4A, B show that this is the case in our expression system as well. Specifically, GIRK currents evoked by application of 5-HT were largely not subject to desensitization when GRK3 and β-arrestin were not expressed (Figure 4A), while a robust desensitization occurs in most oocytes when these two proteins are co-expressed. (Figure 4B; see cumulative results Figure 4C). Examining the effect of Arg220Leu mutation on this signaling pathway we found that the desensitization in oocytes co-expressing the mutant together with the β-arrestin and GRK3 is much weaker in oocytes expressing the mutated receptor than that measured from wt receptor-expressing oocytes. Specifically, while I_5-HT_ declined to 48.6% ± 20.1% of its maximal amplitude after 150 s application of 5-HT in wt receptor, it declined only to 82.6% ± 15.9% of its maximal amplitude in the mutant receptor. This level of desensitization is similar to that measured in oocytes that do not express GRK3 and β-arrestin (82.1% ± 10.2%; co expression of GRK3 and β-arrestin did not significantly affect desensitization; *p* = 0.53). These results suggest that the mutation in Arg220Leu impaired the ability of the receptor to activate the β-arrestin pathway.

**FIGURE 5:**
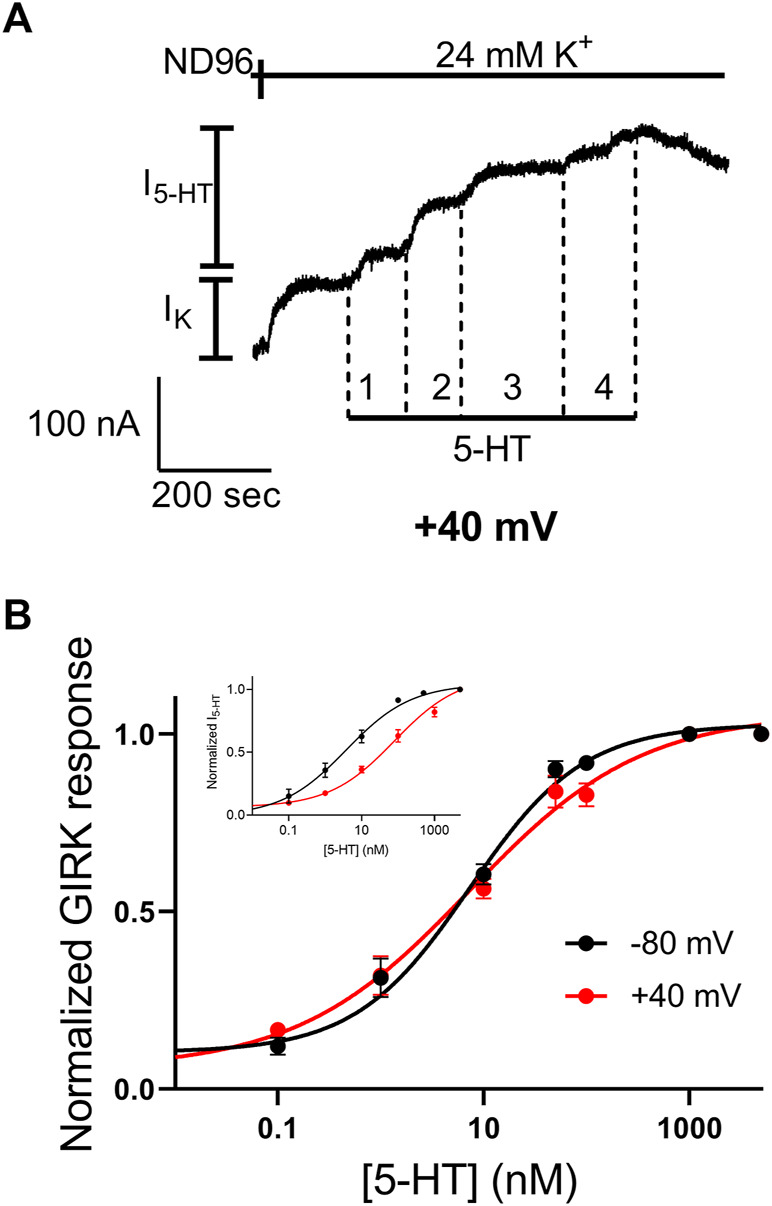
The Arg220Leu receptor is voltage insensitive **(A)** A representative recording of measurement of the relationship between 5-HT concentration and Arg220Leu receptor activated GIRK currents at +40 mV. Basal GIRK current evolved following replacement of the solution to a high K^+^ solution. Then, four different 5-HT concentrations were applied (0.1, 1, 10, 100 and 5,000 nM, numbered 1-4) and the response for each concentration was measured **(B)** Dose response curves for Arg220Leu receptor at −80 mV (black; Taken from Figure 2) and at +40 mV (red). The responses were normalized to the response evoked by 5,000 nM 5-HT at each holding potential. Each point represents the mean (±SEM) from 11-21 oocytes. The solid black and red lines were generated by fitting equation 1 to the data (see Materials and Methods). The EC_50_ values obtained for the two graphs were not significantly different (EC_50(-80 mV)=_6.7 nM and EC_50(+40mV)=_10.2 nM *p* = 0.29). The inset shows the dose-response curves obtained from wt receptor (taken from Tauber and Ben-Chaim, 2022).

We have shown before that the 5-HT_1A_ receptor exhibits voltage dependent activation by 5-HT (Tauber and Ben Chaim, 2022). In some other voltage dependent GPCRs it was suggested that the third intracellular loop of the receptor, and specifically its N-terminal, play a role in this voltage dependence (Ben-Chaim et al., 2006; Ben-Chaim et al., 2019; Ohana et al., 2006; Rozenfeld et al., 2021). Since Arg220 is located in that region, we hypothesized that this mutation may affect the voltage dependence of the receptor. To test this hypothesis we examined the voltage dependence of the mutated receptor. This was done by constructing dose response curve, as described above (Figure 2), also at depolarizing membrane potential of +40 mV. Figure 5A shows an example of recording where four 5-HT concentrations were applied sequentially at +40 mV. From measurements conducted from oocytes from seven batches a full dose response curve was constructed (Figure 5B) and the curve obtained at +40 mV was compared to the curve obtained at −80 mV (Figure 2B). The results show that while 5-HT has lower potency in activating the wt 5-HT_1A_ receptor at +40 mV (Figure 5B, inset; taken from (Tauber and Ben Chaim, 2022)), membrane potential has almost no effect on the potency of 5-HT in the mutant receptor. Specifically, the EC_50_ values were 6.7 nM at −80 mV and 10.2 nM at +40 mV. (The two values are not significantly different *p* = 0.29). Interestingly, the potency at both membrane potentials were similar to that observed at −80 mV in the wt receptor. These results suggest that Arg220Leu plays a crucial role in the depolarization-induced shift in affinity of the 5-HT_1A_ receptor.

**FIGURE 6:**
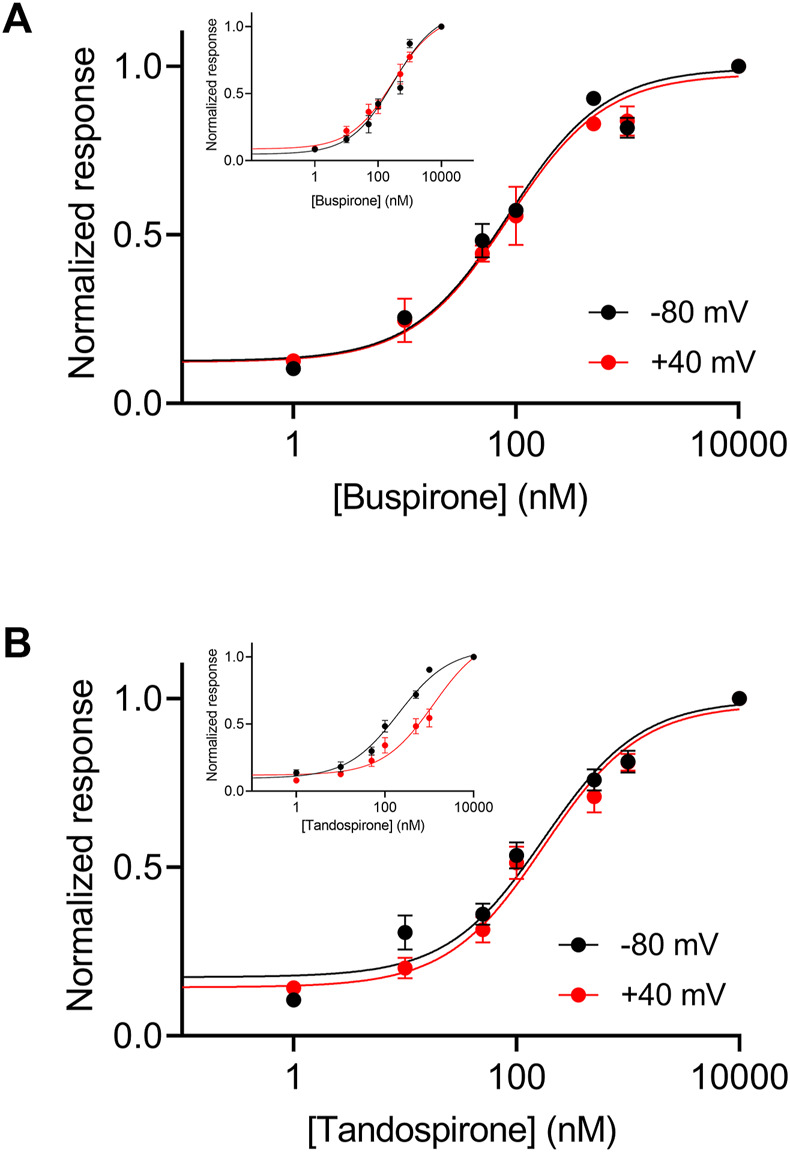
Voltage dependence of the activation of the Arg220Leu 5-HT_1A_ receptor by buspirone and tandospirone **(A)**. Dose response curves for the activation of the 5-HT_1A_ receptor by buspirone at −80 mV (black) and +40 mV (red). Each point here and in **(B)** represents the mean (±SEM) from 12-32 oocytes. The solid black and red lines were generated by fitting equation 1 to the data (see Experimental procedures). The EC_50_ values obtained for the two graphs were not significantly different (EC_50(-80 mV)=_75 nM and EC_50(+40mV)=_97 nM; *p* = 0.15). **(B)**. Dose response curves of tandospirone activated 5-HT_1A_ receptor. The EC_50_ values obtained for the two graphs were not significantly different (EC_50(−80 mV)=_153 nM and EC_50(+40mV)=_211 nM *p* = 0.22). Corresponding curves from wt receptors are shown in the insets in **(A, B)** (Taken from Tauber and Ben-Chaim, 2022).

In the wt receptor, the voltage dependence was found to be agonist dependent. The potency of the agonist tandospirone was shown to exhibit weaker voltage dependency, while the potency of the agonist buspirone is voltage independent (insets in Figures 6A, B) (Tauber and Ben Chaim, 2022). We thus examined the voltage dependence of these agonists. Figure 6 shows that the potencies of both agonists to the mutated Arg220Leu receptor were voltage independent. Specifically, the EC_50_ of buspirone was 74.4 nM at −80 mV and 96.6 nM at +40 mV (not significantly different; *p* = 0.15). The EC_50_ of tandospirone was 153.3 nM at −80 mV and 211.8 at +40 mV (not significantly different, *p* = 0.22). These results are consistent with the role of the Arg220Leu residue in the voltage dependence of the receptor. The results suggest that this role is agonist independent; as the potency of all three agonists tested, were voltage independent in this mutant. 5-HT_1A_ receptors are preferentially coupled to Gi/o proteins to inhibit adenylyl cyclase and can also activate inwardly rectified potassium channels, thus mediating hyperpolarization of the cell (Carhart-Harris and Nutt, 2017).

## Discussion

5-HT and the receptors it activates play an important role in numerous brain functions. Hence, it is not surprising that impairment and dysregulation of the serotonergic system has been implicated in many neurological and psychiatric conditions (Roth, 1994; Berger et al., 2009). The 5-HT_1A_ receptor is the most highly expressed 5-HT receptor in the brain. Several genetic polymorphisms were identified within the 5-HT_1A_ receptor. One of these polymorphisms is a rare mutation in position 220 in the third intracellular loop of the receptor. As this region was reported to play a role in effector coupling, as well is in the voltage dependence of other GPCRs, we sought to investigate the functional effects of this polymorphism. That was done by mutating the 5-HT_1A_ receptor and expressing the mutated receptor in *Xenopus* oocytes. We found two main functional effects of this mutation: 1. Impaired desensitization, probably due to impairment of β arrestin dependent signaling. 2. Diminished voltage dependence of activation by 5-HT and other agonists.

Previous functional study found that the Arg220Leu variant is normally expressed in HEK293 cells (Brüss et al., 2005). Furthermore, this variant did not change the binding properties of 5-HT. On the other hand, the same study found that the ability of the mutated receptor to inhibit cAMP accumulation induced by forskolin was decreased by 60%–90%, suggesting an impairment in G protein activation. The results from our study are consistent with the lack of effect on the ligand binding affinity or expression level in the mutated receptor. However, we found only a mild, not statistically - significant, effect of the mutation on G protein signaling, measured using downstream activation of GIRK channels. This apparent inconsistency may suggest that the effect of this mutant is pathway-dependent. This is in line with the apparent impairment in desensitization observed here (see below). Alternatively, the different observations may be a result of the different kinetic context between the two assays. In the current study, the effect of receptor activation was measured continuously in a tens of seconds time-frame. The measurements of on the other hand, were conducted over a much longer time frame. Recent study has demonstrated that the kinetic context may be a significant factor in measuring different signaling pathways activated by GPCRs (Klein Herenbrink et al., 2016).

Another well-studied signaling pathway in GPCRs is the β arrestin mediated signaling pathway (Violin and Lefkowitz, 2007). Our results demonstrate that desensitization of the receptor following activation by 5-HT, observed only in the presence of GRK3 and β arrestin, was impaired in the Arg220Leu variant, as weaker desensitization was observed in the mutated receptor. Such selective signaling reported here may suggest that the Arg220Leu variant is a biased receptor. A more detailed study of different signaling pathways is needed in order to fully characterize the activity spectra of this variant. Recent studies suggested that the differential effects of 5-HT_1A_ receptors expressed at different synapses might be a result of activation of different signaling pathways (Newman-Tancredi et al., 2022; Powell et al., 2022). Thus, identifying a biased variant of 5-HT_1A_ receptor could lead to a continuing *in vivo* studies aimed for better understanding of the differential roles of the 5-HT_1A_ receptor in the brain and their relation to psychiatric condition, such as depression. That might be of a particular interest as this variant was previously suggested to be associated with major depression (Haenisch et al., 2009).

Voltage was shown to affect numerous GPCRs (Mahaut-Smith et al., 2008; David et al., 2022), including the 5-HT_1A_ receptor (Tauber and Ben Chaim, 2022). Several lines of evidence, suggested that the coupling of G protein to the receptor plays a role in the voltage dependence the M2R (Ben-Chaim et al., 2003; Ben-Chaim et al., 2006; Ben-Chaim et al., 2019; Rozenfeld et al., 2021). The results of the present study, suggesting that a residue in a region implicated in G protein coupling is also crucial for voltage dependence is in line with these findings. The exact role of this region in voltage dependence is not known yet. However, based on the location of this region, outside the electric field of the membrane, it is not likely that it serves as the main voltage-sensing element in the receptor. Indeed, mutating residues in a similar region of the M2R, while abolished the voltage dependence, did not affect the charge movement-associated currents of the receptor, suggesting that these residues do not participate in voltage sensing (Ben-Chaim et al., 2006). We hypothesized that this region may have a role in linking the movement in the voltage-sensing region to changes in affinity (Ben Chaim et al., 2013; Ben-Chaim et al., 2019). It is worth noting that in other GPCRs, such as the alpha-1 adrenergic receptor, no effect of G protein coupling on the voltage dependence was found (Rinne et al., 2013), suggesting that different GPCRs may use different mechanisms of voltage dependence.

Voltage dependence of GPCRs was demonstrated to play a role in several physiological processes, including controlling neurotransmitter release (Parnas and Parnas, 2010; Kupchik et al., 2011) and shaping the excitability of atrial cells (Moreno-Galindo et al., 2011; Moreno-Galindo et al., 2016; Salazar-Fajardo et al., 2018). Moreover, a recent study conducted in D. *melanogaster* revealed that type A muscarinic receptor exhibits voltage dependence binding of ACh and that mutating residues in the third intracellular loop of the receptor abolished the voltage dependence. Strikingly, a fly strain with the same mutations showed impaired learning behavior, suggesting that the voltage dependence is crucial for such a behavior (Rozenfeld et al., 2021). It is thus possible that similar voltage dependence-abolishing mutations in other GPCRs, as the one reported in the current study, will have robust consequences on similar vital brain functions.

## Data Availability

The original contributions presented in the study are included in the article/supplementary material, further inquiries can be directed to the corresponding author.
